# Regulatory role of NFAT1 signaling in articular chondrocyte activities and osteoarthritis pathogenesis

**DOI:** 10.32604/biocell.2023.030161

**Published:** 2023-11-08

**Authors:** MINGCAI ZHANG, TANNER CAMPBELL, SPENCER FALCON, JINXI WANG

**Affiliations:** Harrington Laboratory for Molecular Orthopedics, Department of Orthopedic Surgery, University of Kansas Medical Center, Kansas City, USA

**Keywords:** Osteoarthritis, Chondrocyte, NFAT1, Transcription factor, Regulation of gene expression

## Abstract

Osteoarthritis (OA), the most common form of joint disease, is characterized clinically by joint pain, stiffness, and deformity. OA is now considered a whole joint disease; however, the breakdown of the articular cartilage remains the major hallmark of the disease. Current treatments targeting OA symptoms have a limited impact on impeding or reversing the OA progression. Understanding the molecular and cellular mechanisms underlying OA development is a critical barrier to progress in OA therapy. Recent studies by the current authors’ group and others have revealed that the nuclear factor of activated T cell 1 (NFAT1), a member of the NFAT family of transcription factors, regulates the expression of many anabolic and catabolic genes in articular chondrocytes of adult mice. Mice lacking NFAT1 exhibit normal skeletal development but display OA in both appendicular and spinal facet joints as adults. This review mainly focuses on the recent advances in the regulatory role of NFAT1 transcription factor in the activities of articular chondrocytes and its implication in the pathogenesis of OA.

## Introduction

As the most common joint disease, osteoarthritis (OA) is characterized clinically by joint pain, stiffness, and deformity with radiographical evidence of joint space narrowing due to a loss of articular cartilage, osteophyte formation, subchondral bone sclerosis, and subchondral cyst formation (Buckwalter and Martin, 2006; Lawrence et al., 2008). Current treatments, including pharmacologic therapy, local intra-articular injection, and surgical interventions mainly target OA symptoms, and have only a limited impact on altering the course of OA progression (Richmond et al., 2010; Kloppenburg and Berenbaum, 2020).

The pathogenic mechanisms for OA are not fully understood, especially the mechanisms governing articular chondrocyte activity and triggering articular cartilage breakdown (the major hallmark of the disease) remain unclear. Therefore, a nuanced understanding of the regulatory mechanisms underlying OA pathogenesis is needed for developing effective treatments. Given that many risk factors and regulatory mechanisms have been proposed for the development of OA, in this minireview, we will focus on recent advances in the role of nuclear factor of activated T cell 1 (NFAT1) in the regulation of articular chondrocyte activities and OA pathogenesis.

### The nuclear factor of activated T cell transcription factor family and its transcriptional signaling

**FIGURE 1.**
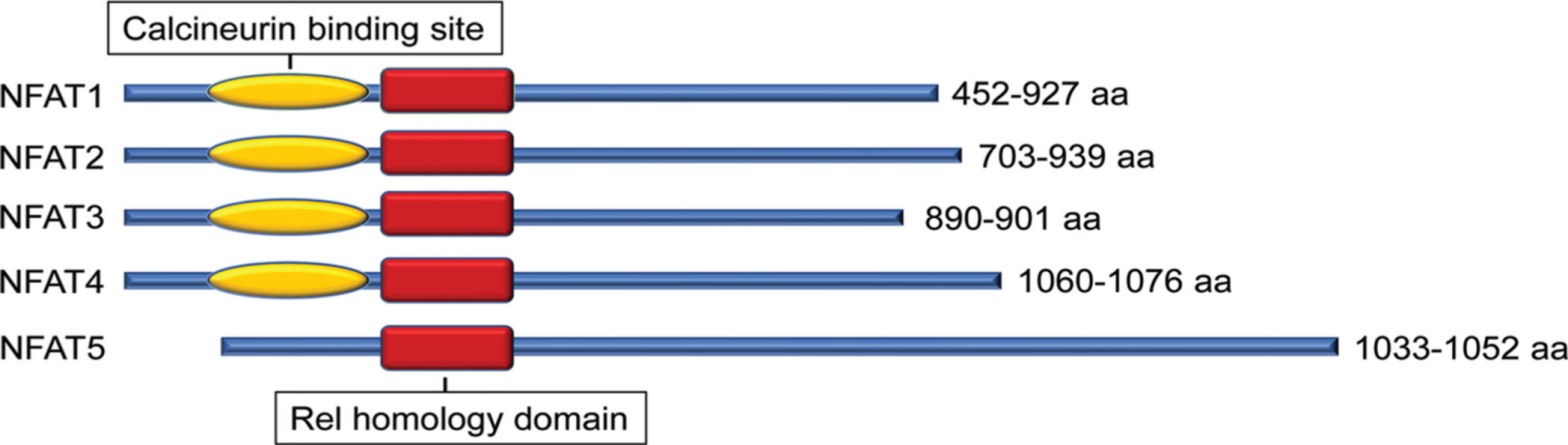
Protein structural character of mouse nuclear factor of activated T cell (NFAT) family members. All NFAT members share the Rel homology domain. Except for NFAT5, NFAT1–4 have a calcineurin-binding domain. The protein size range of amino acids (aa) among different transcript variants is presented.

The nuclear factor of activated T cell (NFAT) is a family of transcription factors first identified in T cells and well-studied for their function in the immune response. There are currently five known members: NFAT1 (NFATc2, NFATp), NFAT2 (NFATc1, NFATc), NFAT3 (NFATc4), NFAT4 (NFATc3, NFATx), and the distantly related NFAT5 (TonEBP, OREBP). These five NFAT members are structurally characterized by a highly conserved DNA binding domain extending beyond the NFAT family to the larger Rel family of transcription factors, including NF-κB (Lin et al., 2013). This highly conserved Rel homology DNA binding domain results in the binding of each of the 5 NFAT family members to the same DNA sequence [(A/T) GGAAA] (Jain et al., 1995) (Fig. 1).

**FIGURE 2.**
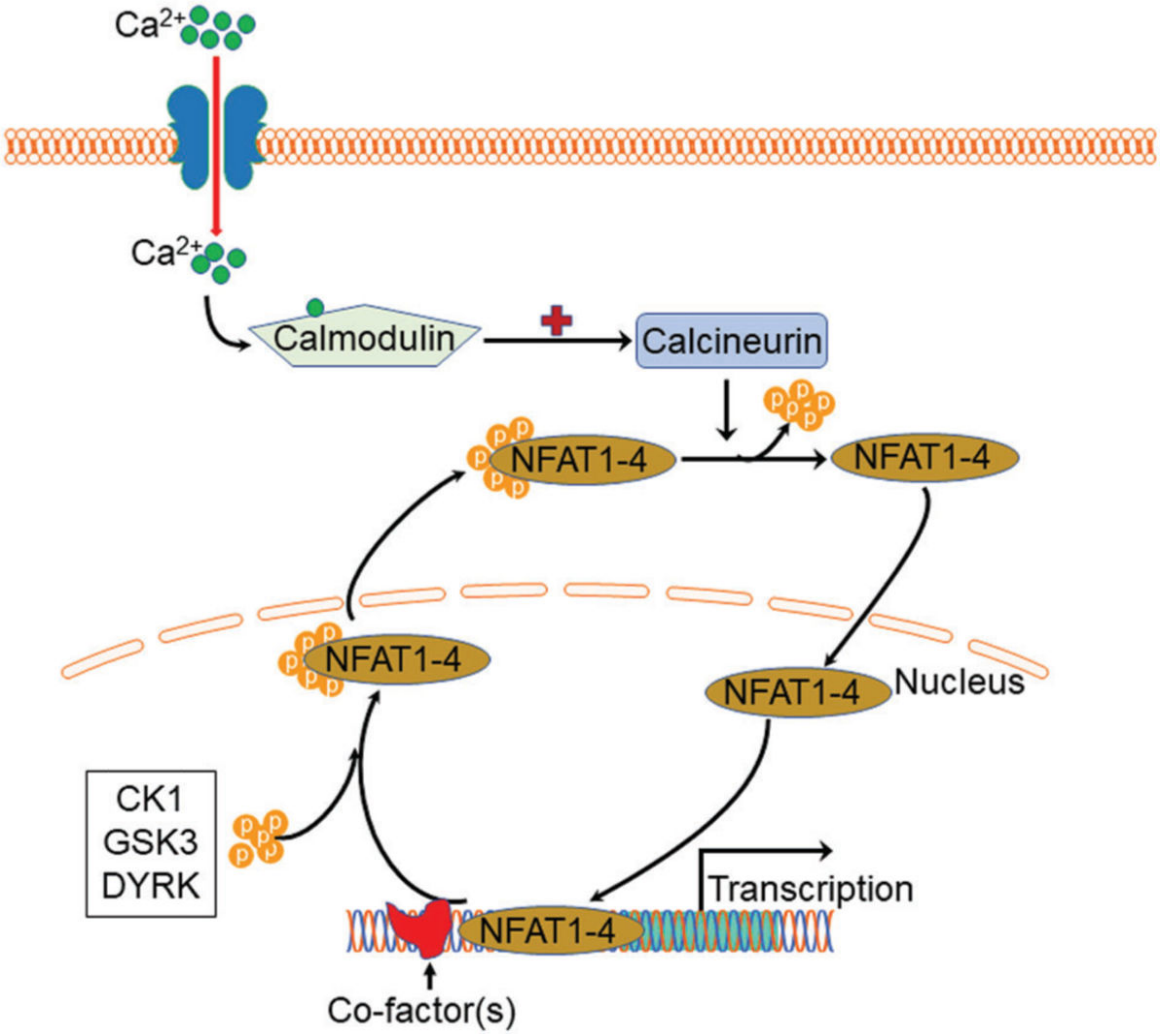
Schematic view of Ca^2+^-NFAT1–4 signaling pathway and phosphorylation cycle. Upon stimulation with an intracellular influx of Ca^2+^, calcium sensor protein calmodulin activates calcineurin which then binds to the N-terminal regulatory domain of nuclear factor of activated T cell 1 (NFAT)1–4 and rapidly dephosphorylates NFAT1–4 proteins, resulting in nuclear translocation. Once the regulation of gene expression completes, NFAT1–4 are re-phosphorylated by casein kinase 1 (CK1), glycogen synthase kinase 3 (GSK3), and dual-specificity tyrosine phosphorylation-regulated kinase (DYRK), enabling NFAT1–4 to be transported back to the cytoplasm.

Except for NFAT5, all four other canonical members (NFAT1–4) are activated by calcium-calcineurin signaling (McCaffrey et al., 1993; Northrop et al., 1994; Pan et al., 2000; Hoey et al., 1995; Masuda et al., 1995; Esensten et al., 2005) as NFAT1–4 have calcineurin binding domain at their N-terminal. In resting T cells, NFAT1–4 proteins are heavily phosphorylated and reside in the cytoplasm. Upon cell stimulation with an intracellular influx of Ca^2+^ that binds to the calcium sensor protein calmodulin, the serine/threonine phosphatase calcineurin is activated. Calcium ionophore ionomycin and polycystin-1 (PC1) can enhance the process of intracellular influx of Ca^2+^, thereby activating the calcineurin/NFAT signaling (Brignall et al., 2017; Puri et al., 2004). Activated calcineurin binds to its binding site located in the N-terminal regulatory domain of NFAT1–4 and rapidly dephosphorylates the serine-rich region and serine/proline repeat consensus sequences (SP-repeats) which are also present in the N-terminus of the NFAT1–4 proteins. This dephosphorylation of NFAT1–4 results in conformational changes that promote their translocation into the nucleus, wherein they regulate gene expression in concert with different cofactors, such as AP-1 (Macián et al., 2001), c-fos (Huang et al., 2006), Jun (Ikeda et al., 2004), GATA4 (Xia et al., 2000), and MEF2 (Blaeser et al., 2000). Once the regulation of gene expression in response to the stimulation is completed in the nucleus, NFAT1–4 are re-phosphorylated by nuclear resident protein kinases, including casein kinase 1 (CK1), glycogen synthase kinase 3 (GSK3), and dual-specificity tyrosine phosphorylation-regulated kinase (DYRK) (Okamura et al., 2004; Arron et al., 2006; Gwack et al., 2006), enabling NFAT1–4 to be transported back to the cytoplasm across the nuclear envelope (Fig. 2). In contrast, the NFAT5, known as a central regulator of cellular response to ambient hypertonicity, lacks docking sites for calcineurin and therefore is regulated by osmotic stress (Miyakawa et al., 1999) (Fig. 1).

NFAT1–5 control the differentiation of specific cells, thereby affecting the formation of specific tissues or organs. For instance, NFAT1 may be involved in adipogenesis and myogenesis (Abbott et al., 1998; Ho et al., 1998). NFAT2 has been found to play a pivotal role during osteoclastogenesis (Takayanagi et al., 2002) and is required for cardiac valve formation (de la Pompa et al., 1998; Ranger et al., 1998). NFAT3-deficient mice were viable and fertile and showed no major macroscopic or microscopic abnormalities after 36 months of observation (Graef et al., 2001). NFAT4 mutant mice exhibited skeletal muscle hypoplasia, reflecting impaired embryonic muscle development (Kegley et al., 2001). Moreover, mice doubly mutant for the NFAT3 and NFAT4 genes had defects in vascular patterning and angiogenesis, indicating that NFAT4 is more important than NFAT3 for cardiac growth and function (Graef et al., 2001). Besides its involvement in the osmotic stress response (López-Rodríguez et al., 2004), complete loss of NFAT5 function showed late gestational or perinatal lethality, whereas partial loss of NFAT5 function resulted in lymphoid hypocellularity and impaired antigen-specific antibody responses in viable heterozygous animals (Go et al., 2004). This review focused on the role of NFAT1 in the regulation of articular chondrocyte (the joint cartilage cell) activities and NFAT1 deficiency in the pathogenetic mechanisms of OA.

### Regulation of articular chondrocyte differentiation and articular cartilage formation

Cartilage cells are derived from mesenchymal progenitor cells during skeletal development. The cartilage cells in the primary and secondary ossification centers are replaced by newly formed bone through the endochondral sequence of ossification. This type of cartilage is called temporal or replacement cartilage (Goldring et al., 2006). In contrast, cartilage cells close to the surface of growing long bones divide and differentiate to form hyaline articular cartilage, a permanent or persistent cartilage because it will not be replaced by bone (Eames et al., 2004; Pacifici et al., 2005). Many factors are involved in the regulation of articular chondrocyte differentiation and articular cartilage formation, although the precise mechanisms of these processes still need to be fully understood.

Sox9 is a pivotal transcriptional regulator essential for articular cartilage formation. Inactivation of the Sox9 gene in mouse limb buds before mesenchymal condensations results in a complete absence of cartilage formation. Mouse embryos in which Sox9 is deleted after mesenchymal condensations exhibit chondrodysplasia and defective joint formation (Bi et al., 1999; Akiyama et al., 2002). Our study in mice found that the Sox9 gene and protein are highly expressed in the early-stage developing articular cartilage at embryonic day 16.5 (E16.5). Sox9 expression was slightly lower but still at a higher level at 1 month of age, at which time the articular cartilage structure is well defined but is still in the growth stage (late-stage developing articular cartilage). The expression of Sox9 is significantly reduced from 2 months of age when the articular cartilage development is completed and then kept at a lower level throughout the life (Zhang et al., 2016). This spatiotemporal expression pattern of Sox9 further reveals the essential role of Sox9 in articular chondrocyte differentiation and articular cartilage formation during joint development.

The ERG (Ets-related gene) transcriptional activator in the *Ets* gene family of transcription factors may also regulate articular chondrocyte formation. ERG is expressed during joint formation and also persists once the articular layer has developed. A C-1–1 variant of ERG has been found misexpressed in developing chick limbs, which imposes a stable and immature articular-like phenotype in the entire limb chondrocyte population, blocking chondrocyte maturation and hypertrophy as well as the process of endochondral ossification (Iwamoto et al., 2000). There is a close spatiotemporal expression of both ERG and growth and differentiation factor-5 (GDF5) in mouse embryo joints. These results indicate that ERG is one of the molecular mechanisms driving the differentiation of immature chondrocytes into permanent articular chondrocytes, exerting its influence through interactions with GDF5 (Iwamoto et al., 2007).

Many other factors, such as matrix metalloproteinases (MMPs) (Bertram et al., 2009), vascular endothelial growth factor (Yin and Pacifici, 2001), fibroblast growth factor (Haque et al., 2007), transforming growth factor β (Joyce et al., 1990), bone morphogenetic protein (Kobayashi et al., 2005) and the Wnt signaling pathway (Macsai et al., 2008), also contribute directly or indirectly to the process of articular cartilage formation.

Early studies reported that NFAT1 may control cellular differentiation programs in organ systems unrelated to the immune system, particularly in adipogenesis and myogenesis (Abbott et al., 1998; Ho et al., 1998). More recently, we found that *Nfat1*^−/−^ mice displayed normal skeletal development, including articular cartilage and joint formation (Rodova et al., 2011), suggesting that NFAT1 is not critical for the formation of articular cartilage and other joint structures.

### Role of nuclear factor of activated T cell 1 in the maintenance of articular chondrocyte activity and articular cartilage homeostasis

Although NFAT1 is not required for articular chondrocyte differentiation and joint formation, it is required for the functional maintenance of differentiated articular chondrocytes in adult mice. Our recent studies have demonstrated that NFAT1 expression in murine articular chondrocytes was undetectable at embryonic day 16.5 (E16.5) and postnatal day 1, but was high at the young adult stage and then declined as the animals aged (Rodova et al., 2011; Zhang et al., 2016). These findings explain why NFAT1 deletion does not affect articular chondrocyte formation during development. In contrast, high expression of NFAT1 in articular chondrocytes of young adult mice indicates that NFAT1 is needed for the function of articular chondrocytes in adults’ Thus, NFAT1 deficiency in adult mice severely impaired articular chondrocyte function, causing subsequent OA onset with abnormal expression of numerous anabolic and catabolic genes, such as *Acan*, *Col2a1*, *Col11a1*, *Col10a1*, *Mmp1a*, *Mmp13*, *Admts5*, *Timp3*, *IL-1b*, and *IL-17a*, etc., in articular cartilage (Rodova et al., 2011; Wang et al., 2009; Zhang et al., 2016a). Moreover, forced expression of *Nfat1* in articular chondrocytes of 15-month-old mice can reverse aberrant expression of specific anabolic and catabolic genes. However, this rescuable effect was reduced in the chondrocytes of 18-month-old mice, indicating that the therapeutic efficacy of NFAT1 on the dysfunction of aged articular chondrocytes is more effective in younger mice (Wang et al., 2009; Rodova et al., 2011; Zhang et al., 2016a). These results suggest that NFAT1 is a key transcriptional regulator that controls the expression of many specific anabolic and catabolic genes in articular chondrocytes, paramount to articular cartilage homeostasis in the adult stage.

In contrast, the expression of Sox9 in mouse articular cartilage is highest at embryonic and newborn stages and then significantly decreases after the completion of joint development (Zhang et al., 2016a). These findings confirm the importance of Sox9 in cartilage formation and suggest a diminished physiological demand for Sox9 in adult articular cartilage. This notion is supported by the evidence that postnatal inactivation of Sox9 in mouse cartilage resulted in a reduction of proteoglycan content in the articular cartilage without histopathological signs of OA by the age of 18 months (Henry et al., 2012).

Our recent studies have uncovered that the age-dependent expression patterns of *Sox9* and *Nfat1* are regulated by epigenetic mechanisms. These epigenetic changes mainly encompass dynamic changes in DNA methylation, histone 3 lysine 4 dimethylation (H3K4me2, a transcriptional activator), and histone 3 lysine 9 dimethylation (H3K9me2, a transcriptional repressor). In mouse articular cartilage, a low *Nfat1* expression in the early developing stage was associated with increased H3K9me2, a high *Nfat1* expression in the young adult stage was associated with increased H3K4me2, and a spontaneous reduction of *Nfat1* expression in the aged phase was associated with decreased H3K4me2 and increased DNA methylation (Rodova et al., 2011; Zhang et al., 2016a, 2016b; Zhang et al., 2019). A growing body of evidence has revealed the epigenetic mechanisms underlying the aberrant anabolic and catabolic gene expression in OA pathogenesis. As a transcription factor, NFAT1 regulates the expression of a cohort of anabolic and catabolic genes in articular cartilage; thus, NFAT1 might be one of the critical, pivotal molecules responsible for the deleterious epigenetic effect in aged articular cartilage. The epigenetically regulated age-dependent spontaneous reduction of NFAT1 expression in articular chondrocytes of aged mice impairs chondrocyte function and results in age-related joint degeneration (Zhang et al., 2019).

### Deficiency of nuclear factor of activated T cell 1 and osteoarthritis

**FIGURE 3.**
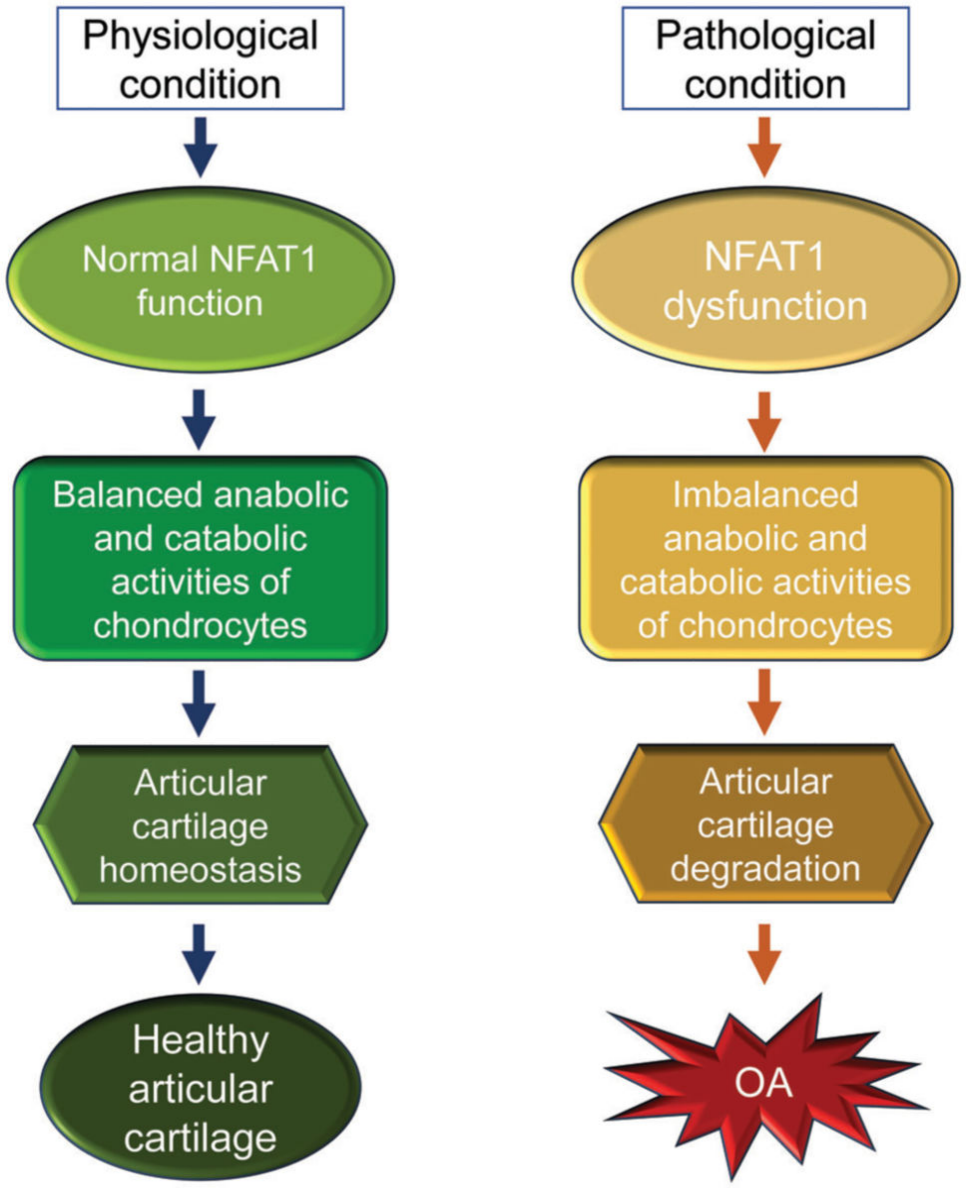
A schematic illustration of the physiological and pathological conditions of nuclear factor of activated T cell 1 (NFAT1) in articular cartilage of mice. In physiological conditions, NFAT1 functions normally by binding its target genes and regulating their expression, resulting in balanced anabolic and catabolic activities of articular chondrocytes, which is essential for articular cartilage homeostasis. In pathological conditions, dysfunction of NFAT1 results in imbalanced anabolic and catabolic activities of articular chondrocytes, leading to articular cartilage degeneration and, finally, the onset of osteoarthritis (OA).

The early reports on the generation and phenotype of *Nfat1* knockout (*Nfat1*^−/−^) mice can be traced back to 1996. Mice with a global NFAT1 null mutation exhibited enhanced immune responses (Xanthoudakis et al., 1996), dysregulated interleukin-4 expression (Hodge et al., 1996), and allergic skin inflammation (Kwon et al., 2016). However, osteoarthritic changes in the peripheral/appendicular joints of *Nfat1*^−/−^ mice were not reported until 2009 (Wang et al., 2009) and in the spinal facet joints until 2022 (Wang et al., 2022). *Nfat1*^−/−^ mice developed normal skeleton but displayed decreased expression of type-II collagen (collagen-2) and aggrecan with increased expression of matrix-degrading proteinases and proinflammatory cytokines in articular cartilage of weight-bearing joints of young adults. These initial changes follow articular chondrocyte proliferation/clustering, progressive articular surface destruction, chondro-osteophyte formation, and thickened subchondral bone exposure, which resembles osteoarthritic changes seen in humans (Wang et al., 2009). NFAT1 deficiency causes aberrant expression of more than 50 anabolic and catabolic genes in articular chondrocytes, resulting in cartilage degradation and OA-like changes (Wang et al., 2009; Zhang et al., 2019; Wang et al., 2022). These studies suggest that NFAT1 is a key transcriptional factor that regulates balanced anabolic and catabolic activities of the articular chondrocytes and overall articular cartilage homeostasis and integrity. In contrast, NFAT1 deficiency or dysfunction may cause imbalance in anabolic and catabolic activities of articular chondrocytes and subsequent cartilage degradation and OA (Fig. 3).

**FIGURE 4.**
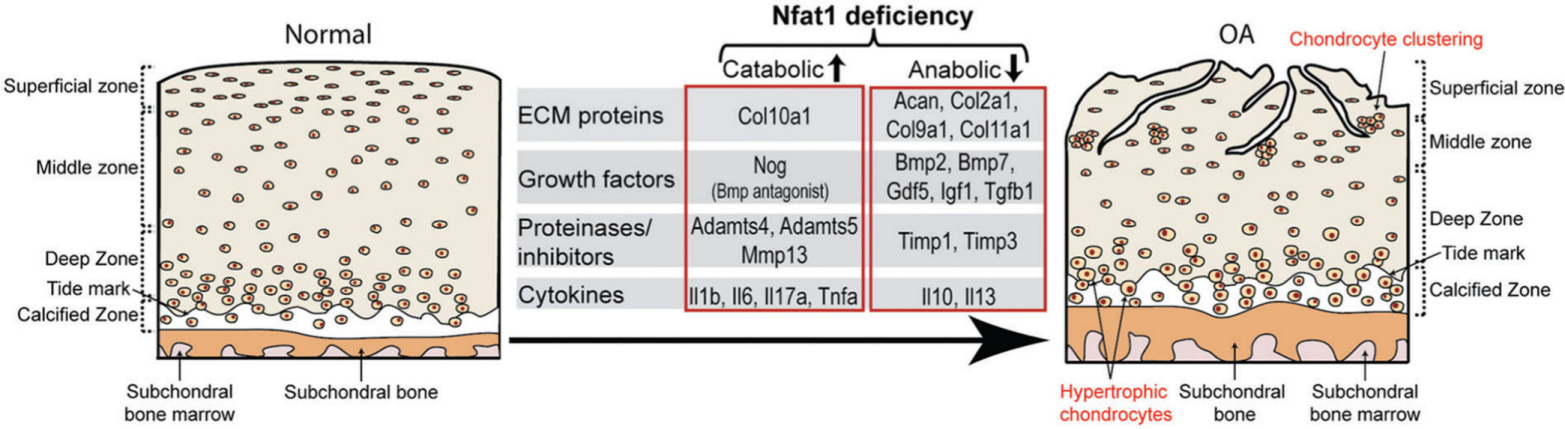
A diagram summarizes the impact of the nuclear factor of activated T cell 1 (NFAT1) deficiency on the expression of specific catabolic and anabolic genes by articular chondrocytes during the development of OA.

The molecular mechanism of NFAT1 deficiency-induced OA was investigated by chromatin immunoprecipitation (ChIP) and promoter activity assays using chondrocytes isolated from femoral head articular cartilage of 3- to 4-month-old wild-type mice or *Nfat1*^−/−^ mice with early hip OA. ChIP assays showed direct binding of NFAT1 to promoters of 21 of the 25 tested genes encoding cartilage-matrix proteins, growth factors, inflammatory cytokines, matrix-degrading proteinases, and specific transcription factors. Promoter activity assays of representative anabolicand catabolic genes showed NFAT1-DNA binding functionally regulated the promoter activity of specific target genes in wild-type chondrocytes but not in *Nfat1*^−/−^ chondrocytes or in wild-type chondrocytes transfected with mutated NFAT1 binding sequences. These studies indicate that NFAT1 protects articular cartilage against degradation by directly regulating the transcription of target genes in these chondrocytes. NFAT1 deficiency causes defective transcription of specific anabolic and catabolic genes in articular chondrocytes, leading to increased matrix catabolism and degradation and destruction of the articular cartilage (Zhang et al., 2019a) (Fig. 4).

A study by Greenblatt et al. (2013) showed that cartilage-specific deletion (via the collagen-2 promoter) of NFAT2 (NFATc1) or NFAT4 (NFATc3) in cartilage displayed no histologic or clinical abnormalities; double deletion of NFAT1 and NFAT4 showed similar histologic and clinical abnormalities observed in NFAT1-deficient mice. However, the mice lacking both NFAT1 and NFAT2 in cartilage displayed severe, spontaneous OA with 100% penetrance, with subluxation of the elbow at about 1 week of age and the metatarsals at an age of about 3 weeks (Greenblatt et al., 2013). Those developmental defects in specific joints were not observed in NFAT1-deficient mice. These findings suggest that NFAT1 is more important than NFAT2 or NFAT4 for the maintenance of articular chondrocyte function and the prevention of spontaneous OA in adult mice.

The epigenetic mechanism is responsible for the up- and down-regulation of NFAT1 signaling and also directly regulates the expression levels of many anabolic and catabolic genes in articular cartilage that are involved in the pathogenesis of OA. In OA chondrocytes, loss of methylation at CpG sites in the promoter region was associated with increased expression of MMP-3, −9, −13, and ADAMTS-4 (Roach et al., 2005; Cheung et al., 2009). Hypermethylated CpG sites in the COL9A1 promoter attenuated SOX9 binding, resulting in the downregulation of COL9A1 expression in OA cartilage (Imagawa et al., 2014). Demethylation of the CpG site at −299 bp of the interleukin (IL)-1β promoter increased the transcriptional response of IL-1β to other inflammatory cytokines in human articular chondrocytes (Hashimoto et al., 2009). Specific and highly expressed long non-coding RNA-CIRs regulate the expression of collagen, aggrecan, MMP13, and ADAMTS-5 in OA cartilage (Liu et al., 2014). Moreover, miRNA-222 regulates MMP13 expression by targeting histone deacetylase 4 during the progression of OA (Song et al., 2015).

## Future Perspectives

This review mainly focuses on the recent advances in the regulatory role of NFAT1 signaling in articular chondrocyte activity and its implication in the pathogenesis of OA. Notably, OA is a whole joint disease involving other joint tissues: subchondral bone, synovium, menisci, joint capsule, and ligaments. Pathological changes in these joint tissues may affect the biological and mechanical properties of articular cartilage. Insufficient recognition of the effect of pathological changes in other joint tissues on OA pathogenesis may negatively affect the efficacy of disease-modifying OA drug (DMOAD) candidates. Therefore, future studies should include the regulatory role of NFAT1 signaling in non-cartilaginous cells and the effects of NFAT1 deficiency on periarticular bone and soft tissues. Additionally, many animal models of OA do not translate into replications in human trials. Further work is needed to investigate if NFAT1 deficiency is involved in the pathogenesis of OA in humans.

In the past three decades, many clinical trials targeting the inhibitor of a single proinflammatory cytokine or proteinase as a candidate DMOAD have been unsuccessful due to insufficient efficacy and/or severe side effects. No DMOAD candidates have been approved by drug regulatory agencies in the United States or other countries (Hellio le Graverand et al., 2013; Kloppenburg and Berenbaum, 2020). These failed clinical trials suggest that inhibition of a single catabolic molecule may not be sufficient for the treatment of OA because the abnormality of both catabolic and anabolic factors is involved in its pathogenesis. NFAT1 is an upstream transcription factor that regulates many catabolic and anabolic factors that are proposed to be involved in OA pathogenesis (Zhang et al., 2019); thus, NFAT1 could be more effective than the previously tested DMOAD candidates targeting a single downstream catabolic or anabolic molecule if it is confirmed to be one of the risk factors for the initiation and/or progression of OA in humans.

## Data Availability

Not applicable.
